# Quantitative imaging assay for NF-κB nuclear translocation in primary human macrophages

**DOI:** 10.1016/j.jim.2007.10.015

**Published:** 2008-01-01

**Authors:** Mahdad Noursadeghi, Jhen Tsang, Thomas Haustein, Robert F. Miller, Benjamin M. Chain, David R. Katz

**Affiliations:** a Department of Immunology & Molecular Pathology, University College London, Windeyer Building, 46 Cleveland Street, W1T 4JF London, UK; b Centre for Sexual Health and HIV Research, Department of Primary Care and Population Sciences, University College London, UK

**Keywords:** Nuclear factor-kappa B, Confocal immunofluoresence, Image analysis, Macrophages

## Abstract

Quantitative measurement of NF-κB nuclear translocation is an important research tool in cellular immunology. Established methodologies have a number of limitations, such as poor sensitivity, high cost or dependence on cell lines. Novel imaging methods to measure nuclear translocation of transcriptionally active components of NF-κB are being used but are also partly limited by the need for specialist imaging equipment or image analysis software. Herein we present a method for quantitative detection of NF-κB rel A nuclear translocation, using immunofluorescence microscopy and the public domain image analysis software ImageJ that can be easily adopted for cellular immunology research without the need for specialist image analysis expertise and at low cost. The method presented here is validated by demonstrating the time course and dose response of NF-κB nuclear translocation in primary human macrophages stimulated with LPS, and by comparison with a commercial NF-κB activation reporter cell line.

## 1 Introduction

Nuclear factor kappa B (NF-κB)/rel represent a family of transcription factors, present in all eukaryotic cells, that regulate inducible expression of wide ranging genes involved in immune responses and cell-cycle regulation. Therefore activation of NF-κB provides a key molecular switch that is relevant to many aspects of cellular immunology research. In immune cells NF-κB is most abundant either as a heteromeric complex of two components, p65 (rel A) and p50, or as a p65/p65 homodimer. The p65 component contains the main transactivating domain responsible for NF-κB transcription factor function. Regulation of NF-κB activity is dependent upon cytoplasmic sequestration in association with an inhibitory molecule, IκBα. As a consequence of intracellular kinase signalling cascades IκBα is phosphorylated, and this leads to its degradation, allowing nuclear translocation of p65/rel A and hence so-called “activation” of NF-κB (Ghosh et al., 1998). Conventional methods for testing NF-κB nuclear translocation utilise a semi-quantitative electromobililty gel-shift assay. This involves incubation of nuclear extracts with ^32^P-labelled oligonucleotides of NF-κB binding sites and separation from unbound probe by electrophoresis in a non-denaturing polyacrylamide gel. However, this assay is principally limited by sensitivity, and requires large scale cell culture (typically > 10 × 10^6^ cells), thus precluding its use with primary cells. Additional disadvantages are the labour-intensive protocol and the need for radioisotopes. Cell lines transfected with NF-κB promoter–reporter gene constructs are used widely to study cell signalling pathways, and are easy to quantitate with colorimetry or luminometry, but are not suitable for most primary cell culture studies. Commercial kits for enzyme linked immunosorbent assays of NF-κB quantitation within nuclear extracts have also become available, but their expense may be prohibitive.

An attractive new strategy is to image NF-κB translocation from the cytoplasm to the nucleus, using immunofluorescence staining. This can be performed on a small scale and at a single-cell level. Fluorescence microscopy, laser scanning cytometry and flow cytometry to quantify NF-κB nuclear translocation have all been reported (Deptala et al., 1998; George et al., 2006; Rogers and Fuseler, 2007; Fuseler et al., 2006). Drawbacks of these previous reports are the need for expensive imaging equipment and/or image analysis software. Here we present an alternative simple method for quantitative detection of NF-κB rel A nuclear translocation which uses standard confocal immunofluorescence microscopy and the public domain Java image processing program, ImageJ. We suggest that the general availability of all three components—fluorescence microscopy, immunostaining reagents, and the analytic protocol-provides a readily accessible method for the study of NF-κB nuclear translocation in primary cell cultures.

For this study we have chosen to use a cell system that has been difficult to analyse previously, primary monocyte-derived macrophages that have been isolated from healthy human volunteers. In vivo macrophages are resident tissue mononuclear phagocytic cells derived from circulating monocytes. They function both as sensory cells of innate immunity, and as effectors, initiating early non-specific host defences, both by local recruitment of other immune cells and by induction of an acute phase response, which leads to systemic priming of the immune system. In vitro work on these cells often includes stimulation by model innate stimuli, now known to be Toll-like receptor (TLR) ligands, such as lipopolysaccharide (LPS) (TLR-4) and Pam_3_CSK4 (TLR-2). Activation of the NF-κB pathway is a common downstream component of the cellular response to many different innate immune stimuli and is used frequently in these cells as a biochemical detection and quantification method to study innate immune cellular activation. Therefore analysis of the NF-κB pathway in macrophages, and in related mononuclear phagocytic cells such as dendritic cells, is an important area of research interest. However, analysis in these cells has been hampered by the lack of suitable methods. Myeloid leukaemic cell lines (e.g. U937, THP-1) are the standard for these experiments, but unless they are very carefully differentiated (which itself may involve NF-κB activation) they are not ideal models. This necessity for an accurate and reproducible primary macrophage system stimulated us to adapt the NF-κB assay as described here.

## 2 Methods

### 2.1 Macrophage culture and innate immune stimulation

Human blood samples were obtained from healthy volunteers. The study was approved by the joint University College London/University College London Hospitals NHS Trust Human Research Ethics Committee and written informed consent was obtained from all participants. Peripheral blood mononuclear cells (PBMC), consisting of monocytes and lymphocytes, were prepared by density-gradient centrifugation of heparinised blood with Lymphoprep™ (Axis-Shield) according to the manufacturer's instructions. PBMC were resuspended in RPMI 1640 with l-glutamine (Gibco, Invitrogen) (NM) containing 5% heat inactivated (56 °C for 30 min) type AB normal human serum (NHS) (Sigma Aldrich) (10^7^ cells /ml) and seeded on to 13 mm (No 1.5) glass coverslips (VWR) using 2 × 10^5^ cells/cover slip. After 1 h at 37 °C non-adherent cells (lymphocytes) were removed by sequential washes with Hanks buffered saline solution (HBSS) (Gibco, Invitrogen). Adherent cells (monocytes) were incubated in NM containing 10% autologous heat-inactivated HS supplemented with 20 ng/mL macrophage-colony stimulating factor (M-CSF) (R&D systems) for 3 days. Any remaining non-adherent cells were removed by further washes with HBSS, and NM/HS was replaced, but without additional M-CSF. This protocol yields adherent macrophages by day six with less than 5% contamination by lymphocytes (data not shown). Ultra-pure LPS (Invivogen) and Pam_3_CSK4 (Invivogen) were used as prototypic innate immune stimuli at different concentrations as outlined below. Polymyxin B (Invivogen) was used as a specific inhibitor of LPS bioactivity.

### 2.2 Immunofluorescence staining

For immunostaining, rabbit polyclonal affinity purified antibody to rel A (C-20) (Santa Cruz Biotechnology) was used (2 μg/ml) with a secondary antibody, Alexa-Fluor (AF)633—conjugated F(ab')_2_ goat anti-rabbit IgG (Invitrogen) used at 4 μg/ml. 10% normal goat serum (Sigma Aldrich) used as blocking buffer, eliminated all non-specific binding of the secondary antibody (data not shown). Cells cultured on glass coverslips were fixed with 3.7% paraformaldehyde (15 min, room temperature) and washed with Tris-buffered saline (TBS). Each coverslip was inverted on to 50 μl of solution placed on impermeable Nesco film (VWR). All reagents were diluted in TBS, and coverslips were washed by immersion into TBS between each staining step. Coverslips were incubated sequentially with 0.2% Triton-X100 (Sigma) (10 min, room temperature), blocking buffer (30 min, room temperature), primary antibody diluted in blocking buffer (overnight, 4 °C) and secondary antibody diluted in blocking buffer (1 h, room temperature). Nuclei were counterstained with 2 μg/ml of the nuclear stain DAPI (Sigma Aldrich) for 5 min. Coverslips were mounted on to glass slides (VWR) using Vectashield hard-set mounting media (Vector).

### 2.3 Image acquisition and analysis

**Fig. 1:**
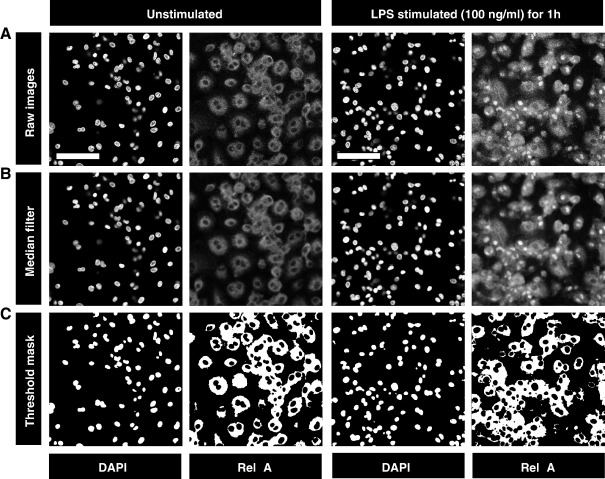
8-bit image files of DAPI and rel A staining are shown for unstimulated and LPS stimulated macrophages (A). Bar represents 100 μm. The sequential processing of these images using ImageJ software is shown to produce binary masks of nuclear and cytoplasmic regions of interest (ROI). A median filter (3 × 3 pixels) is applied to approximate staining to a median value and remove noise (B) followed by automatic thresholding to generate a binary image. The nuclear ROI is defined by the DAPI mask and the cytoplasmic ROI by subtracting the DAPI mask from the rel A mask (C).

**Fig. 2:**
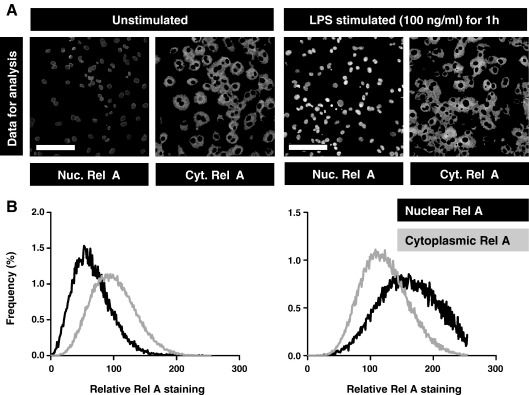
Nuclear and cytoplasmic ROIs are applied to the original NF-κB rel A image files to extract rel A immunofluorescence data for each region in unstimulated and LPS stimulated macrophages (A). Bar represents 100 μm. Histograms of the frequency distribution of fluorescence intensity show increased nuclear:cytoplasmic rel A staining in LPS stimulated cells compared to unstimulated cells (B).

Fluorescence images were captured on a Leica SP2 confocal microscope. DAPI (excitation 405 nm, emission 400–450 nm) and AF633 (excitation 633 nm, emission 650–700 nm) fluorescence were captured using sequential acquisition to give separate image files for each (Fig. 1A). A pin hole of 1 Airy (114.5 μm), scan speed of 400 Hz and 4 frame averaging was used. Photomultiplier tube gain and offset were adjusted to give sub-saturating fluorescence intensity with optimal signal to noise ratio. Image analysis was performed using ImageJ v3.91 software (http://rsb.info.nih.gov/ij). Five high power fields were selected for analysis of each stain. To avoid being biased by the NF-κB staining, each field was selected by viewing nuclear (DAPI) staining only to identify near-confluent cells and thereby maximise the cell numbers included in the analysis. Preliminary experiments demonstrated that this approach provided data on at least 500 cells, and that the variance of the data was not changed by increasing the sample size further (data not shown). For each high power field, binary image masks were created of rel A and DAPI positive staining to define regions of interest (ROI) for analysis. This was done by applying a median filter (3 × 3 pixel radius) to remove noise and to approximate the distribution of staining intensity to a median value (Fig. 1B). Automatic thresholding, using the Isodata algorithm (Ridler and Calvard, 1978) was then used to convert the image to a binary mask (Fig. 1C) that included all fluorescence data above background. The DAPI staining mask was used to define the nuclear ROI. Using the image calculator, the DAPI mask was subtracted from the rel A mask to create a staining mask defining the cytoplasmic ROI. Each of these ROI masks were then applied, by the image calculator, to the original rel A (AF633) staining images to separate nuclear and cytoplasmic staining within each high power field (Fig. 2A). Quantitative fluorescence data were exported from ImageJ generated histograms into Microsoft Excel software for further analysis and presentation (Fig. 2B). Measurement of nuclear fluorescence alone does not distinguish NF-κB nuclear translocation from increased background levels of NF-κB expression or artefactual differences in staining intensity. Therefore nuclear and cytoplasmic staining intensities were compared to give the nuclear:cytoplasmic ratio as a relative measure of rel A nuclear localisation. Nuclear and cytoplasmic histogram data were first normalised for the total number of data points included in the analysis and then comparison was made of the sum of staining intensities. In this way, NF-κB nuclear translocation is represented by an increase in nuclear:cytoplasmic ratio of rel A staining. Multiple images can be batch processed simultaneously in this way by converting individual images into DAPI and NF-κB rel A stacks.

## 3 Data presentation and discussion

**Fig. 3:**
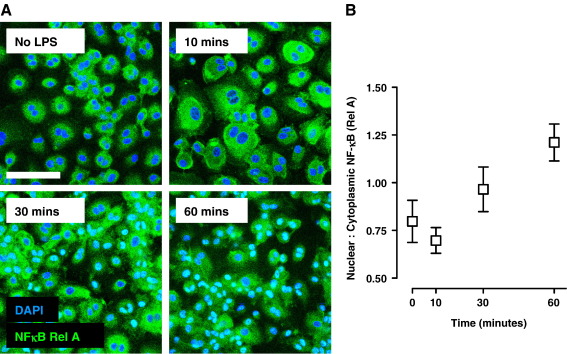
Immunofluorescence staining of rel A (A) and quantification of nuclear:cytoplasmic ratios of rel A staining (B) in a time course study of NF-κB nuclear translocation in LPS (100 ng/ml) stimulated macrophages. Data points represent mean ± standard deviation from analysis of 5 separate high power field images.

**Fig. 4:**
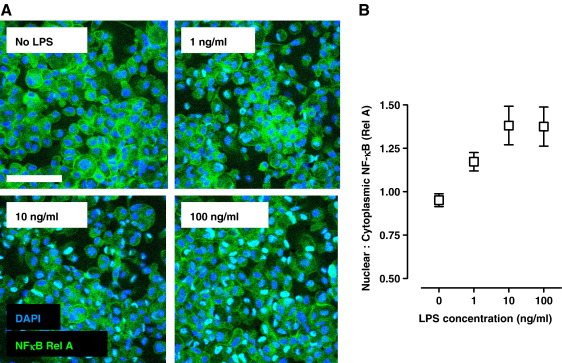
Immunofluorescence staining of rel A (A) and quantification of nuclear:cytoplasmic ratios of rel A staining (B) in a dose response study of NF-κB nuclear translocation in LPS (1 h) stimulated macrophages. Data points represent mean ± standard deviation from analysis of 5 separate high power field images.

**Fig. 5:**
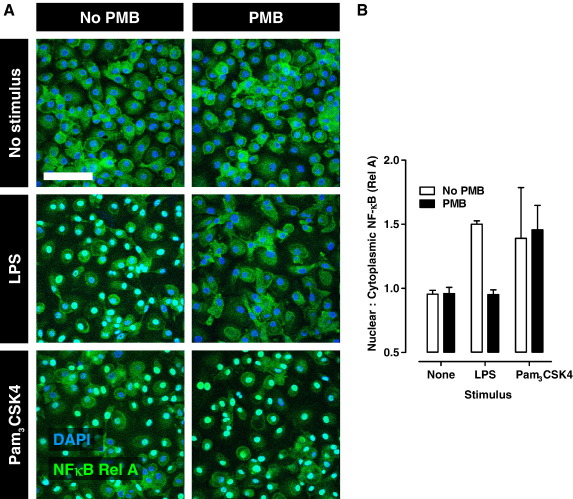
Immunofluorescence staining of rel A (A) and quantification of nuclear:cytoplasmic ratios of rel A staining (B) in unstimulated macrophages and after 1 h stimulation with 100 ng/ml LPS or 100 ng/ml Pam_3_CSK4, with and without 10 μg/ml polymyxin B (PMB). Bar represents 100 μm. Data points represent mean ± standard deviation from analysis of 5 separate high power field images.

To validate this methodology and analysis, time course and dose response studies of LPS induced NF-κB nuclear translocation were performed. Macrophages were stimulated with 0–100 ng/ml LPS in NM with 10% HS for up to 1 h. NF-κB nuclear translocation was evident by 30 min and maximal at 60 min (Fig. 3). The expected increase in nuclear:cytoplasmic ratio was also evident across the LPS dose range (Fig. 4). In addition we tested the effect of Polymyxin B (PMB), a polypeptide that binds and neutralises the bioactive lipid A component of LPS. PMB completely abrogated NF-κB nuclear translocation in response to LPS specifically, and had no effect on stimulation with the TLR2 ligand, Pam_3_CSK4 (Fig. 5).

**Fig. 6:**
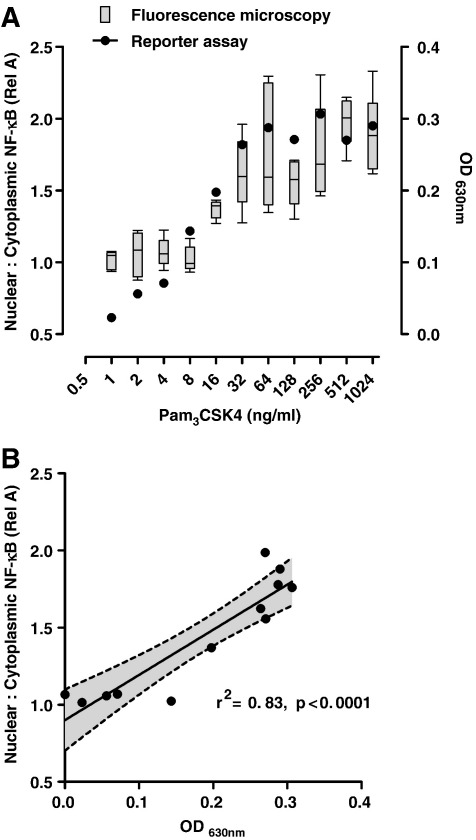
Comparison of NF-κB nuclear translocation by immunofluorescence imaging (nuclear:cytoplasmic rel A staining) and NF-κB activation by reporter gene assay (OD_630_) in TLR-2 transfected HEK-293 cells stimulated with Pam_3_CSK4 (A), shows significant correlation by linear regression analysis (B). Box and whisker plots represent median, SD and range of data from analysis of 5 separate high power field images. Reporter assay data points represent mean of duplicate spectrophotometric measurements. Shaded area of linear regression analysis shows 95% confidence interval.

To further evaluate the quantitation provided by this assay the results were compared with a commercial NF-κB reporter gene assay using the HEK-293 cell line transfected with TLR2 and a secreted alkaline phosphatase NF-κB reporter gene construct (Invivogen). Transfected cells were seeded on to glass coverslips as described above and allowed to adhere for 48 h. They were then stimulated with Pam_3_CSK4 (dose range 0–2 μg/ml) diluted in the manufacturer's detection media. Cells were fixed and stained as above for immunofluorescence staining of NF-κB after 1 h stimulation. Duplicate wells were allowed to incubate for 6 h to allow the reporter gene and substrate reaction to take place. Cell culture supernatants were then harvested to quantify the colorometric reaction spectrophotometrically at 630 nm_._ Quantitative comparison of NF-κB activation using the reporter gene expression assay and NF-κB nuclear translocation by confocal microscopy showed statistically significant correlation (Fig. 6). Importantly, reporter gene expression provides a measure of NF-κB function that is dependent on its nuclear translocation, but not exclusively regulated by it. The difference between measurement of nuclear translocation and NF-κB function is acknowledged and may in part explain the imperfect regression analysis (*r*^2^ 0.83) between the two methods.

The method proposed here is most suitable for adherent cell cultures with relatively large cytoplasmic:nuclear area ratios that allow clear distinction between nuclear and cytoplasmic NF-κB staining. It requires relatively few cells and can be used to study NF-κB nuclear translocation at single-cell level or in mixed cultures. It can be readily applied to the study of NF-κB activation in macrophages, dendritic cells, epithelial and endothelial cells, and fibroblastic cells. We have also been able to apply this method to monocytic cells in suspension (THP-1 and K562 cell lines) by air drying them onto coverslips for immunostaining (data not shown), albeit their typically small nuclear:cytoplasmic area ratio may limit the accuracy of quantitation. This generic image analysis methodology may be applied to quantitative analysis of other transcription factors and signalling events in which assessment of sub-cellular localisation is necessary. Where confocal microscopy facilities are available, this method overcomes the problems related to sensitivity, use of radioisotopes and cost. It can be easily adopted in current cellular immunology research, and given the ready accessibility of the public domain image analysis software, with further validation this methodology may serve as a universal standard that allows better comparison of data from separate experiments and different research groups.
